# Finding the Ionospheric Fluctuations Reflection in the Pulsar Signals’ Characteristics Observed with LOFAR

**DOI:** 10.3390/s21010051

**Published:** 2020-12-24

**Authors:** Leszek P. Błaszkiewicz, Paweł Flisek, Kacper Kotulak, Andrzej Krankowski, Wojciech Lewandowski, Jarosław Kijak, Adam Froń

**Affiliations:** 1 Space Radio-Diagnostics Research Centre, University of Warmia and Mazury in Olsztyn, 10-719 Olsztyn, Poland; pawel.flisek@student.uwm.edu.pl (P.F.); kacper.kotulak@uwm.edu.pl (K.K.); kand@uwm.edu.pl (A.K.); adam.fron@uwm.edu.pl (A.F.); 2 Janusz Gil Institute of Astronomy, University of Zielona Gora, 65-417 Zielona Gora, Poland; w.lewandowski@ia.uz.zgora.pl (W.L.); J.Kijak@ia.uz.zgora.pl (J.K.)

**Keywords:** radio astronomy, LOFAR, pulsar, ionosphere, space weather, GNSS

## Abstract

Pulsars’ signals reaching the atmosphere can be considered being stable under certain assumptions. In such a case the ionosphere remains the main factor distorting signal from the extraterrestrial sources, particularly if we observe them at long radio waves. In this article we present the results of the analysis of relative peak flux changes for two selected pulsars: PSR J0332+5434 (B0329+54) and PSR J1509+5531 (B1508+55), observed with the long radio wave sensor (The PL612 Low Frequency Array (LOFAR) station in Bałdy), together with the analysis of Rate of TEC (ROT) parameter changes measured with the Global Navigation Satellite Systems (GNSS) sensor (IGS LAMA station (IGS: International GSSN Service)). The main objective of the work is to find if the rapid plasma density (observed with the Rate of Total Electron Content (TEC)) has a counterpart in the pulsar observation characteristics. This focuses the attention on ionosphere influence during pulsar investigations at low radio frequencies. Additionally, what was the aim of this work, our results give reasons for using pulsar signals from LOFAR together with GNSS data as multi instrumental ionosphere state probes. Our results show a clear anti-correlation between the ROT and the pulsar profile’s peak flux trends.

## 1. Introduction

### 1.1. Radio Waves Propagation in the Ionosphere

Research on the propagation of radio waves in the immediate surroundings of the Earth began at the turn of the 19th and 20th centuries, with the appearance of the first attempts to use these waves as an information carrier. In the 1930s the first extraterrestrial sources of radio waves were discovered by Karl Jansky [1], which initiated the research of these cosmic signals’ nature. The big development of radio wave research from extraterrestrial sources began in the mid−1950s and continues to this day. The base for the great success of radio astronomy was the huge advance of radio and radar techniques, as well as the introduction of unusual observation techniques that allow for the multiplication of resolution and sensitivity capacities while maintaining the small size of the system’s individual elements. Today these techniques are commonly referred to as interferometry, including large-scale VLBI (Very Large Baseline Interferometry) techniques (see, e.g., [2]).

**Figure 1:**
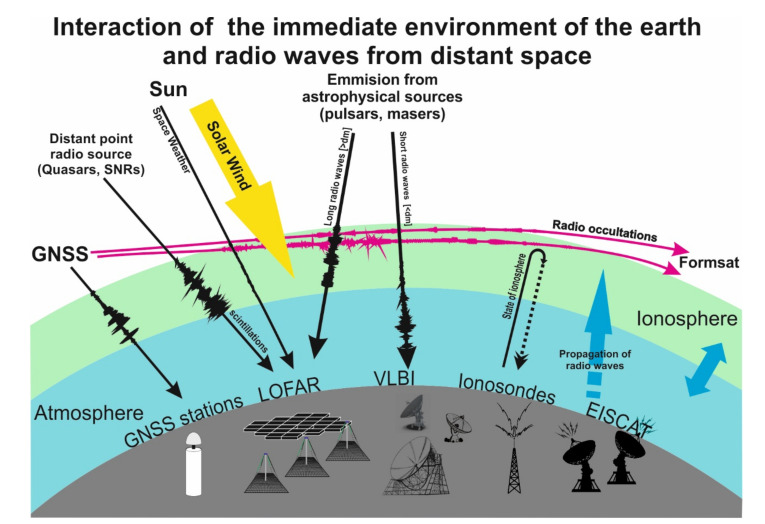
Schematic representation of the interconnectedness of the close environments of the Earth and its research, and its connection to radio waves from distant sources discussed in this document.

On the other hand, radio astronomers quickly realized that the existence of the ionosphere layer has a great impact on the propagation of radio waves in the Earth’s atmosphere, especially in the long-wavelength regime. For centimeter waves, changes caused by minor ionospheric disturbances cause fluctuations in the signal path by up to a dozen centimeters, which changes with a typical timescale of a few milliseconds. This was an evident obstacle blocking the development of radio astronomy on meter waves in the second half of the last century, however today it became an opportunity to implement radio astronomy techniques (as in the 1960s, VLBI methods in geodesy and geodynamics) in the study of propagation of radio signals through the turbulent ionized medium [3,4], as well as the Earth’s ionosphere. This affects the accuracy of currently used geodetic techniques associated with observations of Global Navigation Satellite Systems (GNSS) signals (see Figure 1). These relations were shown already in the concept phase of the LOFAR (LOw Frequency ARay) instrument [5], which is an important part of our work.

However, at high radio frequencies (over 4 GHz) the ionosphere impact on the astronomical radio signals from galactic and extragalactic sources (e.g., supernova remnants (SNR) or quasars) is negligible, the LOFAR operational frequency range (of 10–240 MHz) could be seriously disrupted. The current investigation of the ionospheric scintillations within the bright point radio sources observations was shown by Fallows et al. [6].

On the way from the source to the detection system, radiation encounters the interstellar medium (ISM) (see e.g., [3,4]), an interplanetary ionized matter and the Earth’s ionosphere [7,8]. This gives an incredible opportunity to obtain information on the condition of the ionized medium, including the state and dynamic of the ionosphere. Recent works confirm this state of research completely and show that the precision of ionosphere parameter determination far exceeds that of GNSS-related signal systems (see e.g., [9]).

At some point we come to a situation in which the propagation of radio waves through the ionosphere is not only a stimulus for the study of the ionosphere itself, but also begins to influence the development of other branches of scientific research. A variety of observational infrastructure and research methods have been developed over the last few decades (see Figure 1), the aim of which is to diagnose the ionosphere through the study of radio wave propagation, which perfectly complements the strictly astrophysical observations. In order to study wave propagation a number of radio-based terrestrial systems, such as the European Incoherent Scatter Scientific Association (EISCAT) and the ionosonde networks, have been created. The GNSS satellite networks (Global Navigation Satellite System) and innovative methods related to them and radio occultations (RO) have been initiated or used for research purposes, aimed at better understanding of the ionosphere layers. These include the FORMOSAT-3/COSMIC system (Formosa Satellite Mission 3–Constellation Observing System for Meteorology, Ionosphere, and Climate) and the successor of this system, the currently implemented FORMOSAT-7/COSMIC-2 RO. All these tools provide not only the determination of electron density and TEC (Total Electron Content) parameter, but also a support for numerical forecasts of cosmic weather. The LOFAR system fits perfectly into this trend, which in the LOFAR2.0 phase will be used mainly for purposes related to space weather research. Currently, the LOFAR4SW (LOFAR for Space Weather) project is being implemented under European Union (EU) program Horizon 2020.

The International Validation and Combination Center located at the University of Warmia and Mazury is an important part of the International GNSS Service’s (IGS) Ionospheric Working Group. The Center collects ionospheric data provided by eight International Associated Analysis Centers (CODE/Switzerland, ESOC/Germany, JPL/USA, UPC/Spain, CAS/China, WHU/China, NRCan/Canada and DGFI-TUM/Germany), validates it and combines into final, combined IGS product. Maps provided by SRRC/UWM with high spatial (1 × 1 degree) and temporal (5 min.) resolution will be used in close future by the LOFAR Telescope for ionosphere calibration process.

In our LOFAR pulsar observations we intended to focus on the role of point changes in the state of the ionosphere in a specific direction in the sky that may be caused by various disturbances in the ionosphere, such as different-scale Travelling Ionosphere Disturbances (TIDs) [10,11], plasma bubbles, plasma patches, field-aligned irregularities (FAIs), and down to very small plasma density fluctuations causing radio signal scintillations.

It was also found that the turbulent spatial structure of the ionospheric electron density can be described by a Kolmogorov structure function:

φ_ij_ = (S_ij_/S_0_)^β^, (1)

where φ_ij_ is the phase variance between two points, S_ij_ is the distance between the points, and S_0_ is the diffractive scale at which the phase variance is 1 rad. For index β equal to 5/3 the pure Kolmogorov turbulence occur. At 150 MHz and mid latitudes the standard diffractive scale ranges from 2 and 40 km. The conditions, under which the scale for LOFAR station approach to about 1 km (the Fresnel scale) are called a scintillation. In the case of astronomical research, scintillations are very undesirable because they prevent accurate measurements. This type of disturbance, as well as the TIDs, leads to a change in the observed value of TEC (total electron content) parameters from one to several units [12]. In our case, when it comes to the observation of meter radio waves, this relationship between the point of view of extraterrestrial sources of radio radiation becomes crucial and directly affects the obtained measurement results.

For our specific low frequency LOFAR observations, this physical phenomenon causes changes in the effective antenna beam direction which may cause the pulsar to deviate from the beam center. The change of direction of view Δϕ depends on the wavelength λ and on the TEC like Δϕ = −25·λ·ΔTEC. This causes a drop of the expected telescope sensitivity and in extreme cases (with unusually large TEC values) the source may be outside of the observed telescope beam altogether. All this makes the problem important for astrophysical measurements with telescopes operating on long radio waves. Thus, the main motivation of the authors in the work on the article was to show on the observation sample from the LOFAR telescope, how big is the influence of the ionosphere represented by the Rate of TEC parameter on the long-wave signal from pulsars. 

### 1.2. Pulsars as the Probing Signal Sources

Radio-astronomy observations, due to the specific nature of radiation in the ranges reaching the Earth’s surface, require a very specific approach and specialized tools used for observation. Wilson et al. [2] presented good and detailed documentation for this issue. One of the key elements in the gallery of techniques listed above (GNSS, Formosat-3/COSMIC, and others) is to be implemented and integrated in the IGS Ionospheric Working Group. To use them as well as to apply a new, independent methods of investigations of ionosphere state. From the point of view of long-wave radio astronomy, it is necessary to find a link between the dynamics of the ionosphere and astrophysical objects observation (masers, pulsars, quasars, SNRs). The combination of the already existing ionosphere (satellite) techniques with astrophysical measurements will create a comprehensive tool for ionosphere research with unprecedented precision.

The first pulsar, marked today as PSR B1919+21, was detected in 1967 at the frequency of 81 MHz by [13]. Quite quickly it turned out that a rotating neutron star is responsible for the specific variable and very stable signal. The history of pulsar investigations as well as the methods of observation and pulsar physics is described in detail by [14].

Half a century of pulsar research conducted with the use of radio astronomical methods (both single dish and interferometric) as well as studies in other regimes of the electromagnetic spectrum allowed us to develop a model explaining the properties of pulsar radiation.

Below we briefly explain the key physical properties of pulsars which are relevant for the purposes of our project. Pulsars are rotating neutron stars, which are exotic objects with extremely strong magnetic field (up to 10^14^ gauss) and can rotate up to a few hundred times per second (although a typical pulsar has a rotational period between 0.1 and 1.0 s). These objects are the final products of evolution of massive stars (at least 8 times more massive than the Sun up to about 20 solar masses [14]). The final stage in the life of such stars is a supernova explosion. In extreme conditions during the supernova explosion the protons and electrons merge, forming neutrons. The collapse of interior is halted only when the core shrinks to about 20 km diameter, when the density becomes high enough for the inter-neutron forces to provide an additional source of pressure. The unusual conditions within neutron stars as well as their internal structure are still an area of advanced research. The lack of any possibility of testing the theory of neutron star structure apart from studying them with astrophysical methods results in the existence of several models (see e.g., [15,16]).

The origin of a neutron star from a collapsed stellar core is what causes its extreme properties. The rapid rotation is a simple result of the conservation of angular momentum, and the strong dipolar magnetic field is a result of the compression of stellar one. The closed lines of force of the magnetic field surrounding the neutron star form the so-called “light cylinder”. The magnetic field lines outside this structure may be open [17], because to be closed they would have to travel faster than the speed of light. The region on the surface of neutron star where those open field lines originate is called a “polar cap”. The particles in the magnetosphere emitted from a “polar cap” are accelerated along the open field lines only. The mechanisms of particle emission and acceleration as well as radiation flux formation are still under investigation, see details [18,19,20].

Pulsars’ signal can also be used as tools for other fields of astrophysical research. The first example might be very strong gravitational interactions in close binary systems of neutron stars actually allows for very stringent tests of general relativity. The precise timing pulse periods for binary pulsar PSR B1913+16 confirmed the existence of the emission of gravitational waves [21]. Again, the direct detection of gravitational waves observed as a phenomenon signed GW170817 [22] was related to the merger of two neutron stars.

**Figure 2:**
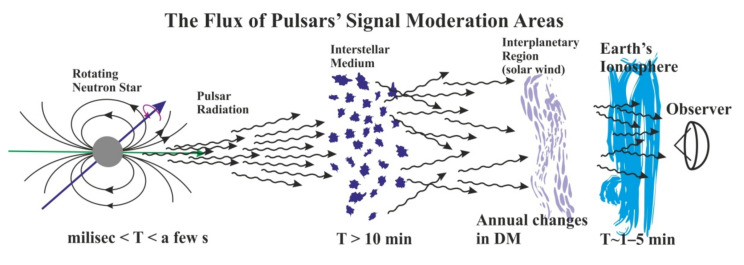
The pulsar radio wave signal is generated in the region of neutron star magnetosphere. The signal propagates through interstellar matter, where the phenomena of dispersion and scattering occur.

**Figure 3:**
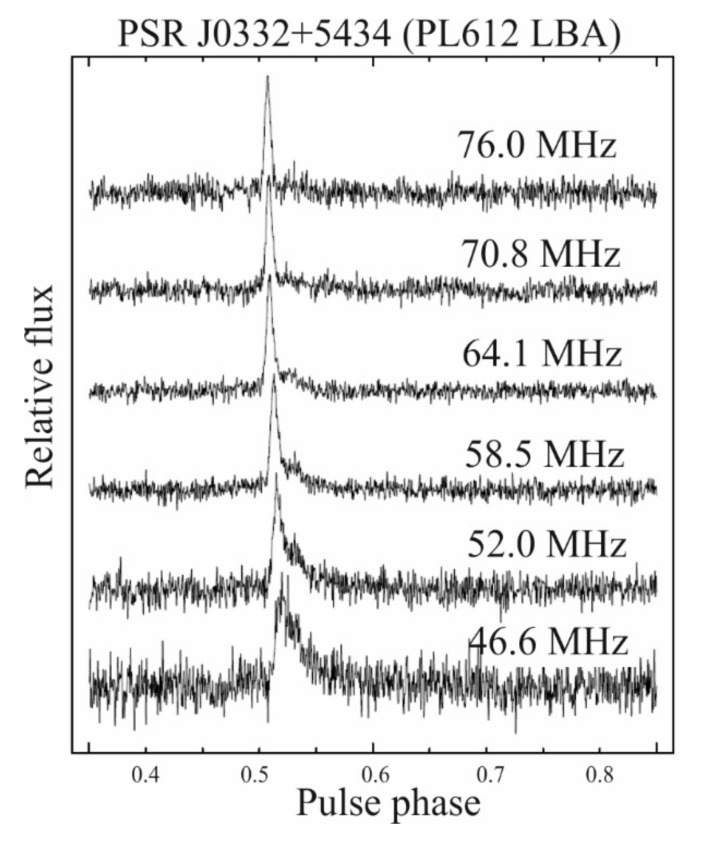
The set of PSR J0332+5434 profiles obtained with Low Band Antennas (LBA) part of single Low Frequency Array (LOFAR) station. The effect of the influence of ionized interstellar matter on the profile with a decrease in frequency is clearly visible.

The pulsating radio signals from neutron stars can be used as probes of the different ionized media, like the interstellar medium (ISM), interplanetary region, or the Earth’s ionosphere (see Figure 2). The pulsar signal propagates through the ISM (neutral atoms and ionized matter). The result of the interaction of radio waves and electrons is the possibility of observing and testing some significant effects affecting the signal reaching the sensors. One of them is the dispersion of the radio waves, which produces frequency dependent delays. This effect is called the dispersion measure (DM) of the pulsar is proportional to the column density of free electrons integrated along the line of sight to the pulsar let us determine distances to pulsars by applying the model of electron distribution in the Galaxy (e.g., NE2001 model [23]). The next two effects observed in pulsars at long radio-waves are the interstellar scattering and scintillation [4]. These effects, clearly visible in the pulsar profiles (see Figure 3**),** are connected to turbulent nature of the ISM and are similar in nature to the twinkling star phenomenon (see for details [24]). Scattering observations show its dependence on the frequency, approximately as ~ν^−4^ [25]. In most models the turbulent nature of the Interstellar Matter is often assumed to have a Kolmogorov’s spectrum of spatial electron density distribution [4].

The observations of pulsars’ signals contribute to the growth of our knowledge about the galactic magnetic fields. Pulsar radio emission is usually highly polarized. The process known as Faraday rotation (θ) occurs when radiation passes through a Galactic magnetic field. The magnitude of the Faraday rotation depends on the wavelength of radiation:

(2) $$\theta =\;\mathrm{RM}\cdot \lambda^{2}$$

where RM is Rotation Measure—a parameter characterizing the influence of the magnetic fields on radio waves traveling from the pulsar to the observer. The designation of Rotation Measure (RM) allows us to estimate the strength of the magnetic field [26].

For the purposes of our observations, the most important aspect of pulsars is that we receive their signals as short pulses of radiation, whenever the neutron star’s radiation beam sweeps our line of sight. Since such a sweep happens exactly once per every pulsar rotation, and this rotation is extremely stable (its regularity is on par with the atomic clocks we use), this allows for relatively easy integration of a signal synchronously with the rotation of a pulsar, which in turn significantly increases the resulting signal-to-noise ratio for these inherently weak astrophysical sources. This allows for the use of pulsar radio signals as a probe of not only the interstellar medium, but also the Earth’s atmosphere (and ionosphere in particular).

The main objective of the work is to find an answer to a complex question. If the low frequency radio astronomical signals are so vulnerable to the ionospheric impact and GNSS techniques are one of the most commonly used tools in ionospheric studies, are there any counterparts between them? Can they work simultaneously with consistent data structure and give consistent, complementary results?

## 2. Observations

### 2.1. The LOFAR Telescope

The LOFAR system was described in detail [27,28]. They showed basics on design, configuration, and the signal processing methods. Hereafter we describe only the most important specification of the telescope and its functions.

The LOFAR interferometer consists of over 100,000 dipole antennas with omnidirectional characteristics. All antennas are grouped into stations. 52 individual stations across Europe were created to this day. The significant number of stations are located in the Netherlands [28], mainly in the region called LOFAR Core, with the Superterp as the central part.

The stations located outside the Netherlands are a part of the International LOFAR Telescope (ILT). Six stations are located in Germany and one station each in UK, France, Sweden Ireland and Latvia. The three Polish stations built by the Polish LOFAR Consortium (POLFAR) Consortium are fully operational from the beginning of 2016 (see details in [29,30]. The LOFAR stations are also planned in Medicina (Italy) and in Rozhen (Bulgaria).

The LOFAR receivers’ bandwidth ranges from 10 MHz to 240 MHz (with a gap in the frequency range from 88 to 108 MHz due to strong Earth-bound interference). Each of the 52 stations is built to be a part of ILT system and interferometric observation mode and consists of two antenna fields. The Low Band Antennas (LBAs) and the High Band Antennas (HBAs). Both antenna fields forming the LOFAR station are described in detail by [31].

**Figure 4:**
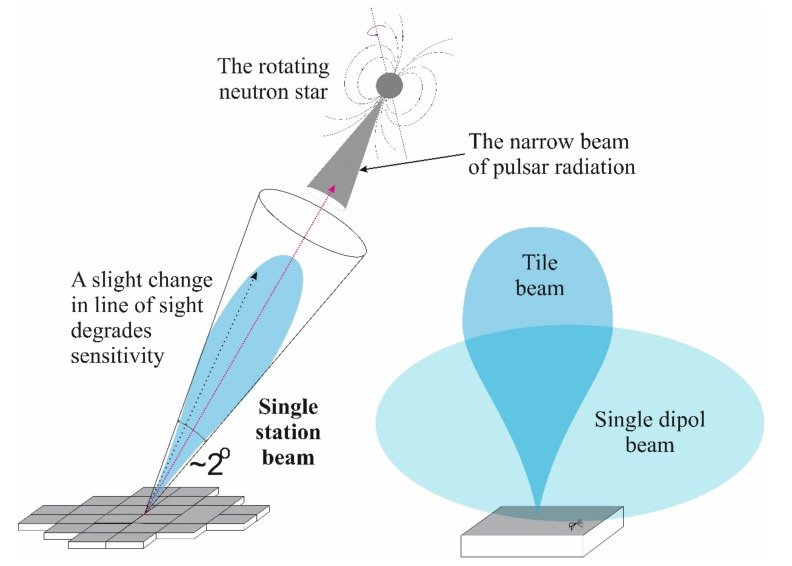
The illustration explains how the individual elements of the LOFAR system “see” sources on the celestial sphere. During the beamforming process, the beam is directed exactly in the direction of the pulsar position determined at the moment of observation. Even a slight change of direction of the line of sight (e.g., by refraction in the ionosphere) reduces the sensitivity of system.

Signals from individual dipoles forming a phase system allow for beam formation (see Figure 4) through digital time delay operations. In this way, a single element of the radio telescope is virtually created, which can be controlled fully electronically. This technique allows to point the telescope at any object in the sky and track its daily movement, and with a suitable computer system it is able to track more than one point on the celestial sphere. The field of view, i.e., the beam width (for HBA parts of single station) is about 2 angular degrees. The ILT instrument (the maximum baseline is currently almost 2000 km) gives an angular resolution close to < 0.1 arcsec at 240 MHz and about 2.5 arcsec at 10 MHz. Resolution of LOFAR is given by αλ/L, where L denotes baseline, α = 0.8 and λ is wavelength in meters [27].

Beamforming is an extremely important process during observation with an instrument such as LOFAR, i.e., a phase antenna. This process is also the basis of the main motivation of our research, which is the study of the pulsar signal distorted by the Earth’s ionosphere. As already mentioned, the basic sensors of the LOFAR system are pairs of dipole antennas. Each of the dipoles in the HBA part has an omnidirectional pattern. 16 pairs of dipoles make a system closed in a single tile, which can create a beam, but it is still wide (see Figure 4, right). In case of observation with HBA system in ILT configuration, for observations around the frequency of 130 MHz, the beam is about 2 degrees wide on the sky. However, the characteristic of the beam (Figure 4, left) makes the sensitivity greatest in the center of the bundle and drops towards its edges. Even slight deviations in the direction in which the position of the source is calculated (and beam is formed) and the real one, changed by refraction in the ionosphere, reduces the sensitivity of system.

### 2.2. LOFAR Signal Processing

The PL612 LOFAR station in Bałdy in the pulsar observations mode see [32] uses a sampled signal from antennas (a clock time/frequency of 5 ns/200 MHz) as the receiver unit input and set band in the selected frequency range. A specially prepared pipeline for pulsar observations described in detail by [31] was also used. In this work the signal from HBA antennas passes through a Polyphase Filter Bank with 512 subbands, each 195.3125 kHz wide. During the process of beam-forming the system is able to create up to 488 beamlets (8-bit mode) with each beamlet acting as a single beam for each subband. Then, the Polyphase Filter Bank uses a 1024 point FFT to divide data stream into 512 subbands. In our observation mode the raw data stream is sent spitted into 4 lanes, 122 beamlets each. Due to the lower sensitivity at the edges of the band for the pulsar observations only three lanes shifted towards the middle of the band are used, starting with beamlet 93, and ending on 459.

In our observations the central frequency was set as 153.808594 MHz and the bandwidth was 71.484375 MHz. This results in a spectral resolution of 0.195313 MHz per spectral channel. A very important step in data reduction is the use of The Digital Signal Processing for Pulsars package (DSPSR) [33] which reduces the raw data to a time series for every spectral channel. Finally, the data is reduced to the full Stokes parameters in TIMER format [33] which is very similar to PSRFITS format [34] and accepted by the PSRCHIVE package. This software allows us to reduce EM interference, combine data previously split into 3 lanes, and prepare data for further measurements, as well as to obtain other useful information (see http://psrchive.sourceforge.net/).

### 2.3. Targets

**Figure 5:**
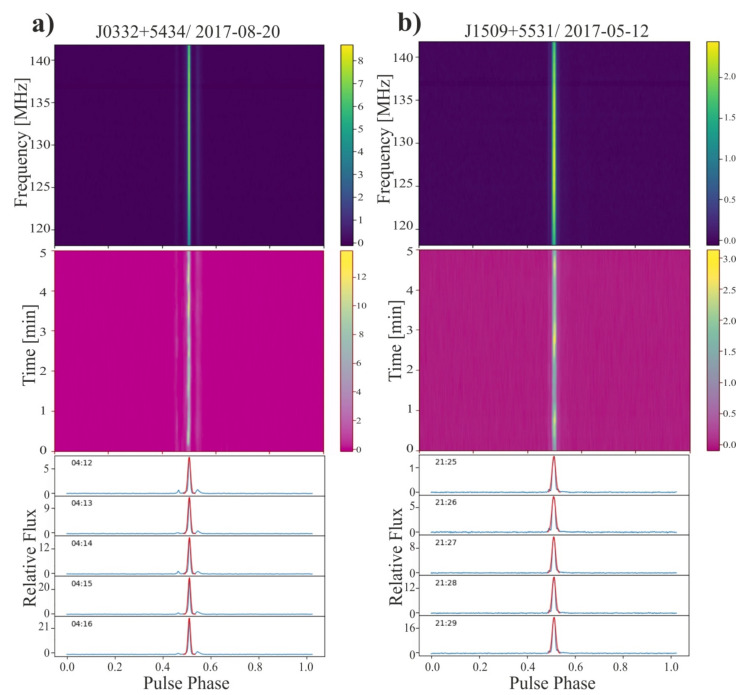
Example observational data for (**a**) J0332+5434 and (**b**) J1509+5531 pulsars from a 5 min observation with 10 s sub-integrations. The upper plots in (**a**,**b**) show the integrated 5 min pulse profile as a function of the observing frequency, the middle plots show the pulsar intensity integrated over the entire frequency range as a function of time. The color bar on the right shows the relative flux. The lower panels in (**a**,**b**) show one-minute integrated pulse profiles with and overlaid Gaussian fit (red) to the pulse shape for both sources.

For the targets of our experiment we have chosen two bright pulsars previously observed with LOFAR (see e.g., [35]). In our previous work we used the data to measurements of sensitivity of the PL612 LOFAR station [31]. Both pulsars were also targets in long term flux density variations study by Esamdin et al. [36]. The examples with visualization of observing data for both observed pulsars, PSR J0332+5434 and PSR J1509+5531, were presented in Figure 5, where the data of the pulsars are shown in two columns, respectively. At the top, panels with dynamic spectra were presented. The spectra for both pulsars in the frequency range from 118 to 142 MHz were shown. The results in this area were cleaned and they are free of EM interferences. The x axis for all panels in Figure 5 is the pulse phase, but interpretation is different in top and middle panels. In the upper panels the signal is split into channels with appropriate frequency width (0.195313 MHz), while in the middle panels the signal is summed over all frequencies, but was shown as a time series of pulses added up every 10 s—i.e., approximately 13 individual pulses were added together in each time channel. In both the top and center panels, the color intensity indicates the signal level. The bottom boxes in Figure 5 present individual profiles in 2D (relative flux vs. profile phase) visualization summed over 1 min each. The relative flux maximum values used in our data analysis were obtained by fitting to the major component of the Gaussian profile. Gaussian curve fittings are marked in red in the bottom sets of profiles presented in Figure 5.

Hereafter we present short notes on individual sources summarized in Table 1.

PSR J0332+5434 (also known as B0329+54) discovered in 1968 [37] is one of the brightest pulsars in the northern hemisphere with the flux of about 1500 mJy (The jansky (symbol: Jy) is a non-SI unit of spectral flux density. 1 Jy = 10^−26^ W·m^−2^·Hz^−1^) at 400 MHz [31,38]. J0332+5434, rotates with a period of 0.71452 s. The distance to the pulsar, the estimation of which is based on the Galactic electron density distribution, was calculated by [36] to be at 1,43 kpc (The parsec (symbol: pc) is an astronomical unit of length used to measure distances. 1 pc ~ 3.086 × 10^16^ m). The DM for this pulsar is 26.7 pc cm^−3^ and the independent of dispersion distance estimation indicates a value of 1.18 kpc [39]. Its observations resulted in an interesting information [40], also suggested possibility of planets existing in his surroundings [41] (this assumption was finally denied). Very important for this and possible subsequent works are the pulsar flux measurements at the frequency similar to LOFAR. The spectra presented in [42] indicate that the 154 MHz flux density is at the level of about 700–1000 mJy. The spectrum presented in [43] suggests flux density between 900 and 1100 mJy and [44] showed the flux density at the frequency of 150 MHz is between 700 and 1000 mJy. In Table 1 we have indicated an average value of 900 mJy with an error of about 15%.

The spectrum of J0332+5434 in frequency range available for HBA part of LOFAR reaches its maximum. The spectral index for high radio frequencies (1.4–23 GHz) mentioned in [42] is −2.2.

PSR J1509+5531 (also known as B1508+55) has the rotation period of 0.74 s and its DM was determined at 19.62 pc cm^−3^ [45]. The calculated model distance [46] is between 1.0 and 1.9 kpc, while new model suggests the pulsar distance of about 2.07 kpc [38]. The flux density measured at the frequency of 400 MHz [38] has been measured as 114 mJy. The value of the flux density at 327 MHz has been estimated at 74 ± 15 mJy few years later [36]. The same authors have also estimated distance to J1509+5531 as 0,99 kpc. The value of the flux density has been determined as 800 mJy ± 15% error (see Table 1).

Spectral data presented in [42] leads to the flux density at the LOFAR frequency range of about 900–1000 mJy which is in good agreement with [44]. Spectral index estimation was α = 2.7 [35] in the frequency region below 100 MHz and α = −2.0 ± 0.1 for the higher frequency.

**Table 1:** Basic information on J0332+5434 and J1509+5531 adapted from ATNF Pulsar Catalogue ^1^ [47].

Name	Right Ascension [h:m:s]	Declination [d:m:s]	Period [s]	Dispersion Measure [pc cm^−3^]	Distance [kpc]	Flux Density ^2^ [mJy]
J0332+5434	03:32:59.368	+54:34:43.57	0.714519699726	26.7641	1.18	~900 ± 15%
J1509+5531	15:09:25.6298	+55:31:32.394	0.739681922904	19.6191	2.07	~800 ±15%

^1^http://www.atnf.csiro.au/research/pulsar/psrcat/. ^2^ Estimated values at 154 MHz based on [31].

### 2.4. The Rate Of TEC from GNSS Observations

In the study we aim to assess the relation between the irregularities in the ionospheric plasma density and recorded variations in the pulsar observations. The Rate of TEC (ROT) is a well-known and commonly-used measure of the TEC variation in time [48]. ROT-derived Rate Of TEC Index (ROTI) describes the occurrence of the radio wave disrupting irregularities in the ionospheric plasma density and is widely-described, very efficient (due to an excellent data coverage) tool in ionospheric studies.

Rate of TEC has also a straightforward correspondence with the plasma irregularities in situ measurements provided by the Langmuir probes from missions like SWARM [49].

ROT can be expressed as:

(3) $$\mathrm{ROT}=\frac{{\mathrm{TEC}}_{i+1}-{\mathrm{TEC}}_{i}}{t_{i+1}-t_{i}}$$

where TEC_i+1_ and TEC_i_ are the TEC values in two subsequent epochs within the continuous arc of the single satellite observation and t_i+1_ and t_i_ are the corresponding epochs of observation. ROT is usually expressed as the change of TEC in the interval of 1 min–in this case unit of the ROT is TECU/min. In the case of single receiver, single satellite and continuous observation arc, the absolute value of TEC is not required for ROT calculation. The transmitter and receiver hardware biases–mandatory to mitigate during the absolute TEC estimation–are cancelling each other during the differential ROT calculation. The TEC value along the line of sight (slant TEC–STEC) can be calculated as follows:

(4) $$\mathrm{STEC}=\left(\frac{L1}{f_{1}}-\frac{L2}{f_{2}}\right)*\frac{f_{1}^{2}{*f}_{2}^{2}}{f_{1}^{2}-f_{2}^{2}}*\frac{c}{K}$$

where L1 and L2 are carrier phase observations performed on frequencies f_1_ and f_2_, c is the speed of light in the vacuum, and K is a constant factor equal to 40.3.

ROTI index is calculated as a standard deviation of the ROT values within the chosen interval. The ROTI-based ionospheric irregularities map for the northern hemisphere are provided as an IGS product [50,51]. ROT and ROTI are successfully used in different ionospheric structures description like plasma bubbles [52] and TIDs [53].

In this study we focus on the raw ROT, as ROT is derived directly from the GNSS radio signal, and can be treated as a measure of the ionosphere-conducted disruption in the signal. Such an approach meets the idea of the distortion in pulsar signals observations—in both cases we observe irregularities impact on signals from the radio sources.

### 2.5. Simultaneous Observations of Pulsars and ROT Towards Them

The basic idea of the simultaneous observations of pulsars and ROT is to observe both pulsar and GNSS signals passing the same region of the ionosphere—considering region as an area narrow enough to catch ionospheric irregularities. In order to achieve this, we have tied each observation with a point within the ionosphere. For the ionospheric irregularities analysis, we have applied a single thin layer ionosphere model. This model assumes that all the ionospheric plasma is concentrated within the infinitely thin layer fixed on the certain altitude for the whole Earth. This simplified approach is commonly used in the ionosphere studies that do not require the information about the 3D plasma distribution, focused only on horizontal plasma distribution.

Assuming a single thin layer model of the ionosphere, for each trans-ionospheric line of sight we can find an ionospheric pierce point (IPP). For each collected pulsar observational data set we have calculated sets of corresponding IPPs. In the first step we simply used the radec2azel function from the Python’s [54] library with the pulsar’s right ascension and declination as well as the coordinates of the Baldy LOFAR station HBA field center point. After obtaining the full set of horizontal coordinates of subsequent 1-min pulsar observations we recalculated those into IPP latitude and longitude:

(5) $$\begin{array}{c} \Psi =\arccos\left(\left(\frac{R_{E}}{R_{E}+h}\right),\cos,E\right)-E\\ {\mathrm{lat}}_{\mathrm{IPP}}={\mathrm{lon}}_{0}+\Psi \cos\mathrm{Az}\\ {\mathrm{lon}}_{\mathrm{IPP}}={\mathrm{lon}}_{0}+\frac{\Psi \sin\mathrm{Az}}{\cos{\mathrm{lat}}_{\mathrm{IPP}}} \end{array}$$

where Ψ is a so-called offset angle, R_E_ stands for the Earth’s radius, h for the ionospheric layer altitude, E for the elevation angle, and Az for the azimuth.

Having the ionospheric pierce points for pulsar observation we searched for the nearby GNSS IPPs. We defined ‘nearby’ as a zone within 3 degrees of the angular distance from the source—such a scale corresponds with the size of medium scale disturbances. This also corresponds with the scale of observable irregularities with the 30 s GNSS observations, which is from 30 km up to hundreds of kilometers [55]. To find the GNSS close-ups, we have collected the Global Positioning System (GPS) and GLObal NAvigation Satellite System (GLONASS) data from the LAMA permanent station of the International GNSS Service (IGS) network. The station is located ~20 km away from the Baldy LOFAR station. However, the ionospheric irregularities can be very small and 20-km range between sensors might cause skipping some of them, in this study we use 30-s averaged data that correspond with larger structures. So long averaging time sets sensitivity threshold on the structure size around few degrees of angular resolution [55]. Moreover, considering LOFAR observing beam of 2-degree width on the sky (so all data within are integrated) and also 3-degree searching window around the source, it is right to assume that GNSS observations performed on the station 20 km away can be still treated as ‘nearby’.

For each epoch of the pulsar observations we have calculated the GNSS IPPs for every satellite in sight. Satellite IPPs calculation was slightly different—we have used the 3D cartesian coordinates of the satellite from the precise orbit files (IGS product). Using again the Pymap3D library (ecef2aer function) we have obtained the horizontal coordinates and calculated the IPP longitude and latitude using same algorithm as previously (Equation (5)).

Searching for the GNSS-LOFAR close-ups required the direct checking the angular distance for each GNSS IPP with the pulsar-observation IPP in each epoch. We calculated the distances as orthodromes and set the searching filter for the value of 3 degrees. For each ‘found’ point we have calculated the ROT value, which was then compared with the relative flux and signal to noise ratio of the LOFAR observations.

**Figure 6:**
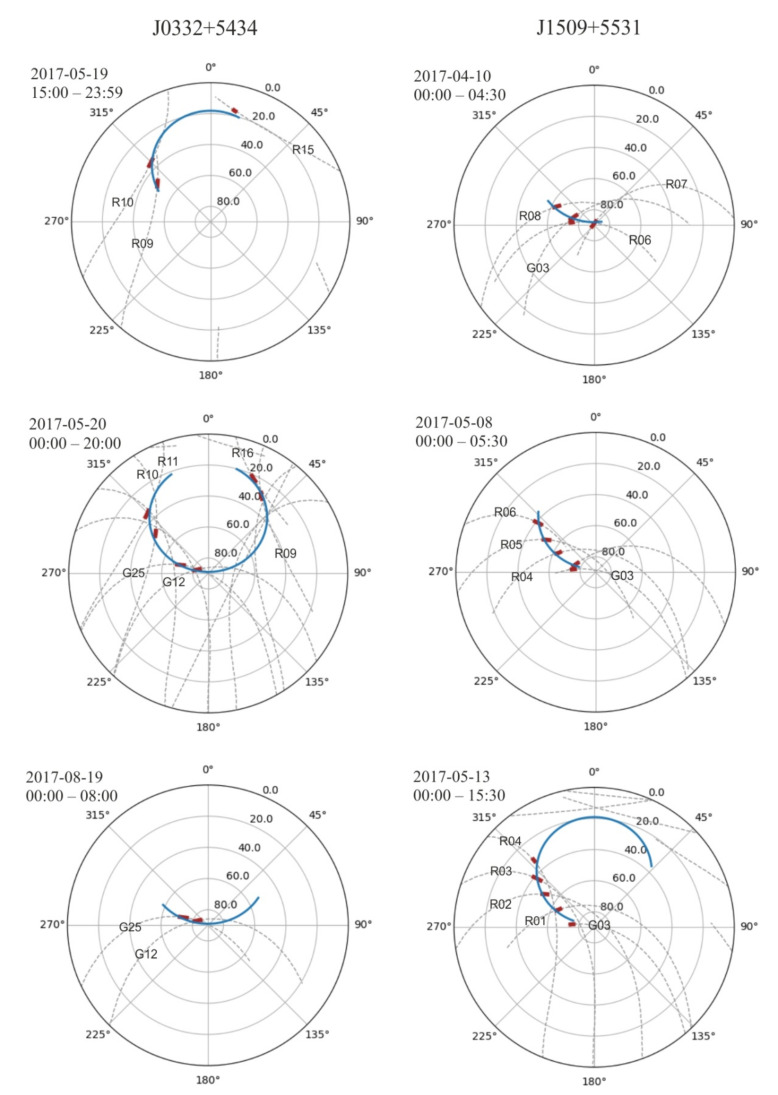
Plots in the azimuth coordinate system in a polar projection presenting changes of pulsars position (blue lines) and Global Navigation Satellite Systems (GNSS) satellites position (dashed lines with satellite number-the letters R and G denotes GLObal NAvigation Satellite System (GLONASS) and Global Positioning System (GPS) satellites, respectively.) in the sky. The red parts of GNSS line show sectors where the angular distance of the satellite and pulsar on the sky was less than 3 degrees.

In the Figure 6 we are showing the mutual position of the trajectory of the satellites and the pulsar in the azimuth coordinate system in a polar projection. The changes of pulsars position (blue lines) and GNSS satellites position (dashed lines with satellite number) in the sky is presented. The letters R and G denotes GLONASS and GPS satellites, respectively. The red parts of GNSS line show these sectors where the angular distance of the satellite and pulsar on the sky was less than 3 degrees. For these moments, the ROT and pulsar relative flux measurements were made. 

## 3. Results

### 3.1. Ionospheric Scintillations Observed with LOFAR PL612 Station

To show the importance of the results, it is worth mentioning that LOFAR stations are a very good tool for observing scintillation of radio waves from distant bright, point sources [7,8,9]. Of course, when observing astrophysical objects, we strive to reduce the influence of the ionosphere as much as possible, however, this is not always a routine activity. On the other hand, monitoring the phenomena connected to ionosphere is one of the observation points in the single station mode for a few LOFAR stations. Although the current observation technology does not allow simultaneous observations of pulsars and ionospheric phenomena, below we present examples of such observations. In studies of the ionosphere, we observe two discrete astronomical radio sources for LOFAR system: Cyg A and Cas A.

Cygnus A (Cyg A) is a radio galaxy, one of the strongest radio sources in the sky. It contains an active galactic nucleus (AGN). The super-massive black hole at the core has a mass of 3 × 10^9^ solar masses. Cassiopeia A (Cas A) is a supernova remnant (SNR) and it is the brightest extrasolar radio source in the sky at frequencies above 1 GHz.

**Figure 7:**
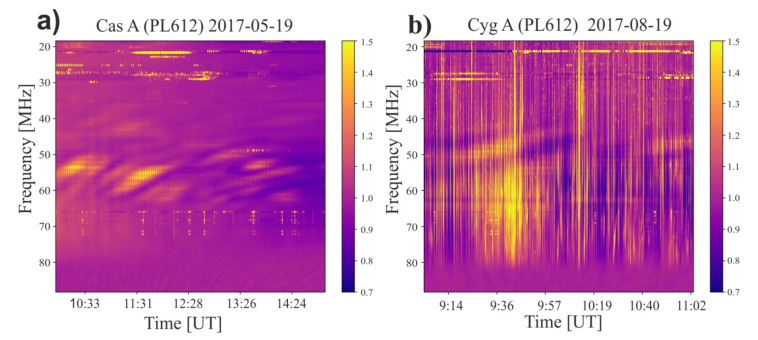
The dynamic spectra for two distant discreet radio sources observed with LBA part of PL612 LOFAR station in Bałdy are presented. (**a**) Cyg A observed 2017.05.19; (**b**) Cas A observed 2017.08.19. For both part Y axis denote frequency and X axis shows the time. Color bars representing relative flux received by the system. The structures visible in the figures show changes caused by the ionosphere.

Figure 7 shows examples of dynamic spectra obtained in the direction of Cassiopeia A (left part) and Cygnus A (right part). Data were taken from LBA part of LOFAR PL612 station and represent frequency span from 18 MHz up to 88 MHz. Temporal resolution is 1 s. The pipeline for this kind of observations is described in detail in [30]. The colors represent relative flux. The slightly stretched structures (left) and vertical structures (right) show how the electron density changes towards the observed sources, which affects the transfer of radio waves through the ionosphere with also taking into account the frequency of the radio wave. Such observations, as they are carried out at lower frequencies, are much more sensitive than the commonly used GNSS methods [3,8].

### 3.2. The Pulsar’s Profile Flux vs. ROT Correlation

Only part of the information collected during the recording of the pulsar signal was used for our analysis. First of all, the frequency range has been narrowed from 118 to 142 MHz (see Figure 3). It was dictated by the need to minimize the impact of disturbances on the relative flux. The observations were conducted in May and August 2017 for J0332+5434, and in April and May 2017 in the case of J1509+5531. The single session observing time was between a few and a dozen hours, and in this time range we were looking for the pulsar line-of-sight close-ups with GNSS satellites. Our limit connected with the 2° LOFAR beam size during chosen observation mode was 3 degrees angle in the sky. The Table 2 and Table 3 show summary information on the observations. The Figure 6 shows all the close-ups with the limitations presented above. Two columns denote observational status on the sky for three chosen for analysis observation epochs for PSR J0332+5434 and PSR J1509+5531, respectively. The blue lines show the pulsar’s path on the celestial sphere as shown in the azimuth coordinate system, in a polar projection. The time of observations is presented in Table 2 and Table 3 for both observed pulsars. The dashed lines show the trajectories of the conjunct GNSS satellites. The fragments of the satellite’s path in the sky where the distance to the pulsars did not exceed our assumed value of 3 angular degrees are bold and marked in red.

**Figure 8:**
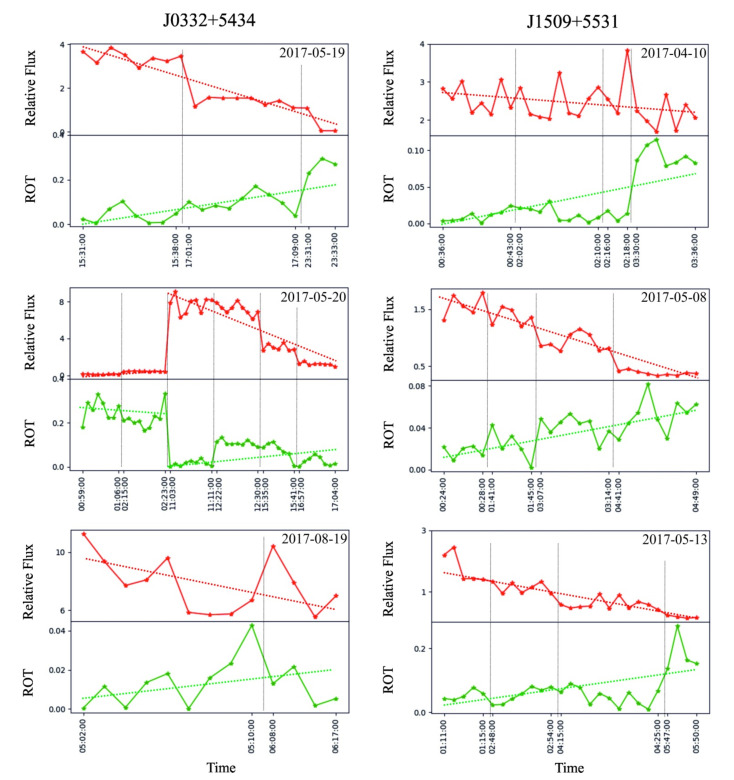
Comprehensive presentation of comparison of results of pulsar profile’s relative flux (upper panels, red lines) and Rate of TEC (ROT) (lower panels, green lines) values taken from GNSS data (see Section 3.1). In each panel, all observation points corresponding to all recorded close-ups of the satellite and pulsar in the sky were collected. Vertical lines separate individual close-ups. Each plot is complemented with a linear trend dotted line.

For further analysis, the results obtained during the close-ups were collected, i.e., the value of the relative flux averaged for one-minute sub integrations and the ROT values calculated with the same period (see Equation (2)). The main motivation was to search for correlations between changes in the state of the ionosphere towards the pulsar and its relative flux. The correlation coefficients were calculated using Pearson method implemented in Python script. The data we collected is shown in Figure 8. The data has been presented in the similar arrangement of panels as before, so that the Figure 8 is consistent with the Figure 6 when it comes to the layout of the presented content in the panels. The relative flux values for the main component of the pulsar profiles were determined (for all previously designated close-ups) with the minute integration time and then by using Gaussian curve fit. Values obtained are presented in red on the top parts of each panel. For each obtained value of the relative flux, the ROT values were calculated (see Equation (3)) and the results are presented in the lower parts of each panel using the green color. Data from all close-ups in a given pulsar observation epoch (see Table 2 and Table 3, columns 1–3) were presented in one sequence. The reason for this is that the correlation applies only to those results that were obtained during the conjunction of the pulsar and the satellite. To be clear, the observation intervals have been marked in vertical tiny dotted lines on each panel, as well as begin and stop of each subsequent was specified on the x axis. Each panel is composed from 2–6 observation intervals corresponding to the satellite-pulsar tracking close-ups (see Figure 5). However, there may occur some random correlation within single observation intervals, the most important correlation information is the inter-dependence of the general trend and interval-to-interval variabilities. This dependence is clearly visible for the J0332+5334 pulsar observed on 20 May 2017 (left middle panel of Figure 7)—one can clearly see a significant increase of the pulsar peak flux during decrease of ROT value. For a better insight to the general trend, each plot is complemented with a linear trend, however the trend line for J0332+5334 pulsar observed on 20 May 2017 (left middle panel of Figure 7) was split into two trends: before ROT jump and after. In this single case the total observation time span was significantly longer and the discontinuity both in ROT and pulsar peak flux was more than evident. The weakest correlation may be noted on the lower left panel of Figure 7 (J0332+5334 pulsar observed on 19 August 2017), the correlation coefficient around −0.14. Such situation is caused by two factors—number of observing points was low (only 13), ROT value was in general very small (maximum below 0.05), the result of which lead to mostly random correlations. That also explains correlation calculation between whole datasets—variability within the short intervals may lead to random, unreliable results. Taking into account situations of higher ROT values and variability, the general trend is obvious—stronger fluctuations of TEC are ‘seen’ in the pulsar profiles as a decrease of the radio flux peak value. The significance of the existence of such an anti-correlation on the typical measured level from −0.46 to −0.74 is considerable (only one value in Table 2 is below of this range). It clearly shows that for certain specific types of observations (long waves and relatively short duration), changes in the measured parameters are closely related to the density of ions in the ionosphere layer. Without taking into account the influence of the ionosphere, our interpretation, which refers to astrophysical phenomena, may lead to false conclusions.

**Figure 9:**
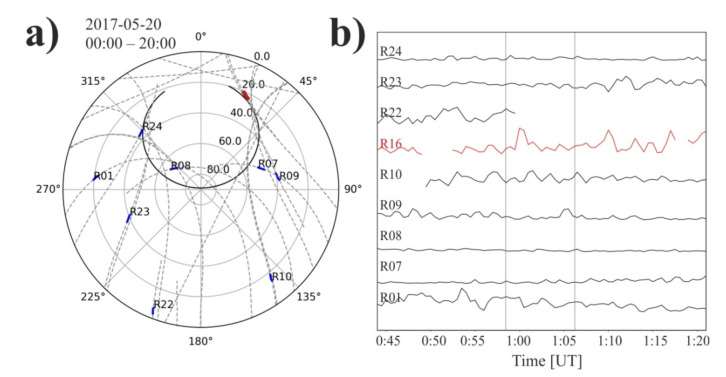
The Example of ROT data for one close-up PSR J0332+5434 and R16 satellite (red bold line) compiling the ROT in the direction of the other satellites observed at the same time (blue lines). The (**a**) is the visibility of GNSS satellites (dashed lines) during observations of PSR J0332+5434 (solid line) presented in the azimuth coordinate system in a polar projection. The (**b**) is the relative ROT value taken from the observation on 20 May 2017, from 0:44 to 1.21 UT. The moment of close-up is indicated by dotted horizontal lines.

In order to check whether the signals from satellites above the horizon in other areas of the sky show significant similarities, we selected satellites visible during our measurements and combined the ROT data with the ROT data from satellites observed towards the pulsar-one observation epoch for each. Data is presented in Figure 9. The left plot is the visibility of GNSS satellites during observations of PSR J0332+5434 with regions, where data was taken from marked in bold. The close-up to the R16 satellite (20 May 2017 from 0:56 to 1:06 UT) is marked in red. The same time for the rest of satellites is marked in blue at their paths. This plot is made in the azimuth coordinate system in a polar projection, what is similar to way of presentation in Figure 6. It is worth paying attention to the right part of Figure 9, where the ROT values towards a set of satellites are presented. We took a slightly wider range of time, and the moment of close-up of the R16 satellite with the pulsar is marked with horizontal dotted lines. This plot clearly shows that the ROT parameter measured in different directions in the sky shows significant differences.

The above considerations and measurements may lead to a clear conclusion that LOFAR is extremely sensitive to even slight changes in the state of the ionosphere towards observation.

The large influence of the ionosphere on the level of the signal reaching the Earth from distant galactic and extragalactic objects such as rotating neutron stars, observed as pulsars is easy to see not only by the anti-correlation between ROT and relative flux shown in the Figure 8.

**Figure 10:**
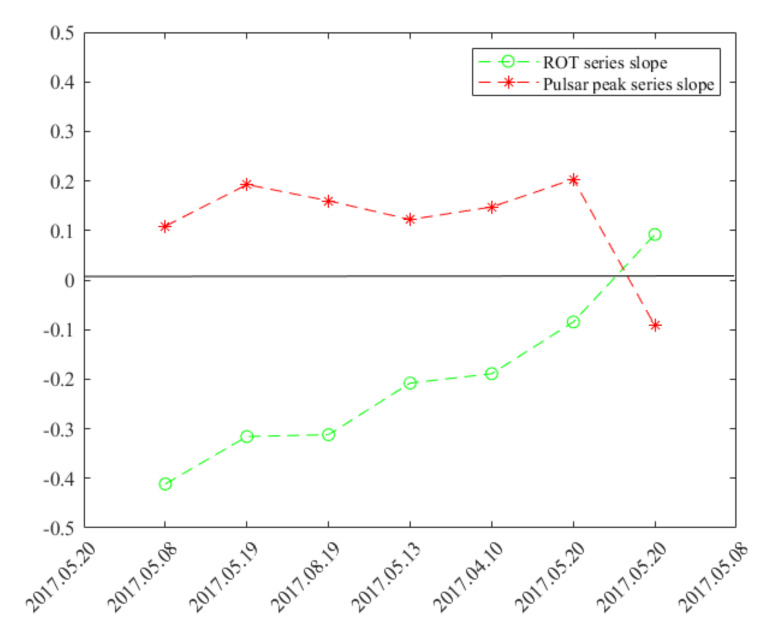
Linear trend slope parameters of the ROT and pulsar peak intensity series.

Figure 10 represents the general trend slope parameter estimated within the ROT and pulsar peak intensity series. Comparison of the slope parameters of the corresponding time-series reveals the ROT and pulsar intensity series anti-correlation in the terms of the general trend. It can be clearly seen that slope parameters for each pair of corresponding series have opposite values—positive slope for ROT series corresponds with a negative slope of the pulsar intensity series and vice versa.

**Figure 11:**
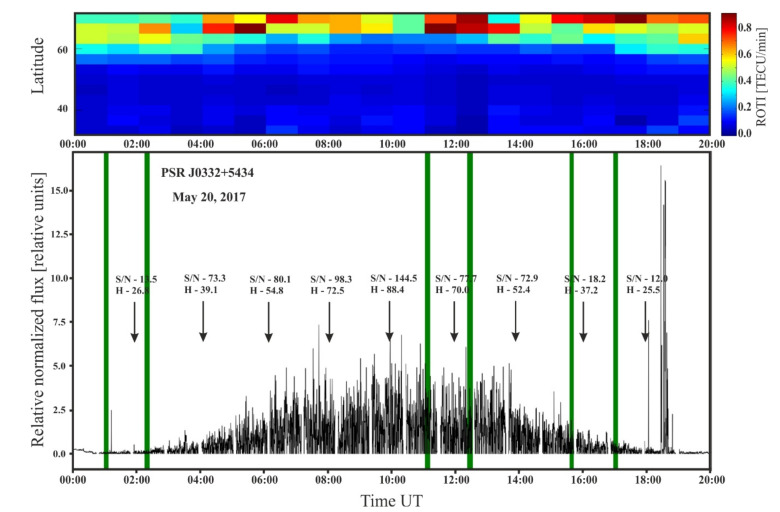
The plot at the bottom part shows fluctuations of relative flux for the entire period of observation of the pulsar PSR J0332+5434 on May 20, 2017. The level of relative flux was normalized. The green lines show close-ups time (see Figure 6 and Figure 9). The height of the pulsar above the horizon-H and the signal-to-noise ratio (S/N) are also noted. Top panel presents the latitudinal extent of the Rate of TEC Index (ROTI) auroral oval during one day.

Figure 11 presents the PSR J0332+5434 peak intensity during the 20-h long observation period (00–20 UT) as well as general behavior of ROTI over the different geographic latitudes. One can clearly see that the pulsar peak intensity is lower during the nighttime and rises after the sunset. On the pulsar plot we have also marked the LOFAR-GNSS close-ups to exaggerate that close-ups cover different phases of the pulsar’s intensity diurnal variation. The height of the pulsar above the horizon and the signal-to-noise ratio (S/N) are also shown in Figure 11. This data has been included to make it clear that flux fluctuations, which are the largest during the day, are not associated with an increased amount of noise associated with the LOFAR sensor characteristic [35]. ROTI colormap in Figure 11 is based on observations collected from the chain of GNSS stations along the meridian of 20° E longitude in order to assess the ROTI diurnal behavior. The auroral ionospheric irregularities oval spreads towards lower latitudes during night [56,57,58]. That ROTI behavior and night-side auroral oval expanse towards lower latitudes corresponds with the diminishing pulsar intensity observed with LOFAR during night. However significant ROTI values reach 60° N, it should be remembered, that ROTI is a quantified index of the nominal ROT behavior (here, a hourly average is presented), and thus smaller fluctuations, such as presented ones, might be smoothed out by the long averaging intervals. ROTI variation should be treated as a background—more detailed comparison of the nominal ROT and peak density are presented above and confirm the anti-correlation.

## 4. Discussion

The meaningful influence of the ionosphere on LOFAR observations was discussed during the development phase of this instrument, when it was pointed out that it could be an independent source of knowledge about this layer of the atmosphere [5]. Studies of the influence of the ionosphere on the observations of astronomical sources have been conducted since the very beginning of the telescope, but they are mainly focused on space weather [7,8,9]. The recent results show the scrutiny of the ionospheric scintillations of the radio signals from the two distant radio sources [3] and present the sensitivity of the LOFAR instrument. We also present an ionospheric scintillation spectrum in the Figure 6 as an example of the LOFAR sensitivity for ionosphere density irregularities.

Pulsar surveys using the LOFAR telescope are conducted either in a special mode, where single station is used for observation [35], as well as the central area (i.e., the LOFAR Core) is used in the mode of creating multibeam and data analysis (see e.g., [34,59,60]). For the majority of pulsar observations conducted with LOFAR the influence of the ionosphere is neglected and treated only as a nuisance, decreasing the sensitivity of the instrument. A large portion of pulsar observing projects does not require absolute flux density measurements (which in fact is not an easy task for LOFAR) and in such cases the estimation of relative flux density variations is more than sufficient [61]. There are also investigations which are using ILT imaging mode [62]. Only the last of the mentioned observation modes during data correlation reduces the averaged influence of the ionosphere.

As we mentioned before, the need for calibration for disturbances in the wave front caused by the ionosphere was taken into account and discussed already in the instrument’s construction phase [63,64], particularly in imaging interferometric mode. However, data obtained in beamforming mode (single station and core) are subject to minimal analysis of the influence of the ionosphere, in most cases, the results obtained after a long integration time (10–15 min and longer) are taken for analysis, which averages the results for distorting the direction of view, and thus shifts the target to the edge of the beam or outside of it, which ultimately causes changes in the recorded value of relative flux of the pulsar profile.

**Figure 12:**
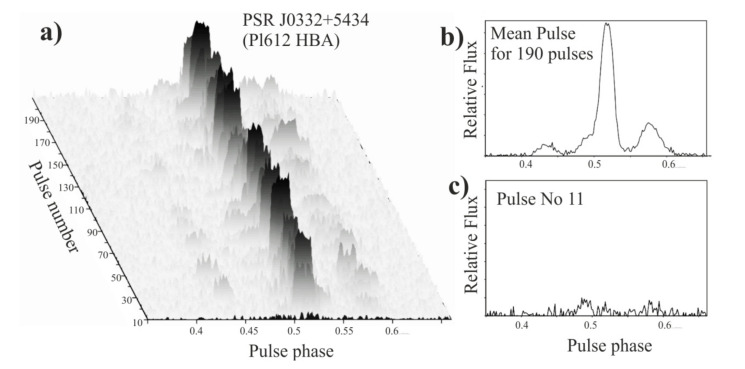
The figure shows individual pulses collected in an isometric view (**a**), which corresponds to data presented in Figure 4. A different method of data reduction was used-not 10 s integrated profiles were taken, but single pulses. In (**b**) panelthe average pulsar profile from adding 190 individual pulses is shown. In the (**c**) part an example of a single pulse is presented.

In Figure 12, in a suggestive way, we show what the danger may be when analyzing the data if we do not take into account the ionosphere influence. While the average profile blurs the influence of signal changes along the way through the ionosphere, in single profiles this influence may be significant. We may come to the point when we are not able to definitely decide whether the changes from pulse to pulse are related to the dynamics of the ionosphere or to the source itself, which is a neutron star. There are, however, a few pulsar observing techniques, where the influence of the ionospheric variations can be very impactful. The most important of these is the single-pulse observing mode, where the incoming pulsar signal is not integrated and a full time series comprising of individual pulses is analyzed. Unfortunately, the analysis of the influence of the ionosphere is not taken into account during the analysis of such observations (see e.g., [65,66]). This is due to the fact that there is usually no external information on the state of the ionosphere during the observation and in the direction of the source. This means that the majority of the observations of several pulsar related phenomena-such as mode switching, subpulse drifting, nulling, etc., are prone to be flawed due to the impact of ionospheric variations.

Knowing how strong the ionospheric fluctuations impact for the pulsar’s signal (particularly observed with a single LOFAR station) are, our intention was to invert the well-known problem of ionospheric calibration to obtain some information about the ionosphere fluctuations in the LOFAR pulsar’s signal. Such an inversion is currently known and performed during the calibration of interferometric observation [28], however until now it is only made for imaging mode of observation, not in a single station beamformed mode, as presented in our study.

In our work we found that ionospheric fluctuations behavior described with widely-used GNSS-based indices such as ROT and ROTI have a clear counterpart in the pulsars’ radio flux intensity registered by LOFAR. We chose the rate of TEC as an ionospheric indicator, as it is commonly used for investigation of different irregularities’ structures within the plasma density. One can note clear correspondence between diminishing pulsar’s peak intensity observed with LOFAR during night and the ionospheric irregularities auroral oval spread towards the mid-latitudes (observed e.g., with ROTI, as in Figure 11). However, that correspondence may be questioned due to the ROTI long averaging time, it finds confirmation in the anti-correlation between ROT values and pulsar’s peak intensity observed during LOFAR-GNSS signal paths close-ups.

Our work is the one of the first to take up this topic and thanks to the tools created, subsequent analyzes of a much larger sample will be conducted in close future. The results obtained in our research encourage us to analyze such effects for a much larger number of pulsars in the near future.

## 5. Conclusions

In our work we present observational data of two pulsars, PSR J0332+5434 and PSR J1509+5531, obtained using the LOFAR station located in Bałdy, designated PL612. This data was confronted with the data obtained by the IGS LAMA receiver located in Lamkówko. Both of these instruments are owned and managed by UWM. In our investigations firstly we searched for all the instances when the satellites of the navigators were closer than 3 angular degrees from the pulsar. Results of this search are presented in Figure 6.

Our main objective was to assess if the LOFAR pulsar observation characteristics performance in face of the ionospheric plasma gradients can be compared with the GNSS TEC fluctuations.

Pulsar data has been prepared and measured for all retrieved close-ups. We used 1-min integrated profiles to determine the maximum relative flux by using of Gaussian fit to main profile component. The one-minute integration time is connected with the physical processes causing the flux variation (Figure 2). For all the moments of relative flux determination we also measured ROT value. Figure 8 shows the corresponding graphs integrated for all the satellites close-ups in the period in which pulsar was observed. Figure 10 also confirms that increasing trend of the ROT values corresponds with the negative trend in the pulsar peak intensity and vice versa.

As a result of our study, we can confirm, that there exists an echo of ionospheric fluctuations (observed with ROT) in a pulsar’s flux intensity registered with LOFAR PL612 station. It is proven by a strong negative correlation between the ROT value and pulsar’s peak flux, the most evident, when looking at jumping values (strong increase of a ROT value corresponds directly with a decrease of the pulsar’s peak flux and vice versa).

The important result of our work was the start work on the creation of a multi-instrumental tool based on LOFAR and GNSS that, independently, associated with observations of extraterrestrial sources of long radio waves, will allow the assessment of the state of the ionosphere. The results obtained in our research can be especially useful for those pulsar observations in LOFAR program which require good control of ionospheric conditions. A system could be created that would search these close-ups. As we showed before, even for longer pulsar observations there will be only a few such close-ups, but even this may allow ionospheric changes to be assessed by the interpolation method. This will allow, at least roughly, to remove the influence of the ionosphere from the observation. The corrections to the pulsar flux measured on this basis could then be applied to the data, significantly improving their quality and, above all, reducing the chance that the ionospheric effects would be taken for some new effect in the pulsar radiation mechanism.

In future works we plan to increase the number of used LOFAR stations, which would allow us to perform more detailed observation of particular ionospheric structures and involving additional instruments such as ionosondes.

## Figures and Tables

**Table 2:** The summary of measuring data for PSR J0332+5434.

Date	Start Time ^1^	End Time ^1^	S/N ^2^	Relative Flux ^2^	ROT ^2^	No of Close-Ups	No of Points	Correlation Coefficient ^3^
19 May 2017	15:00	23:59	131.6	2.08	0.294	3	13	−0.743
20 May 2017	00:00	20:00	184.4	3.41	0.334	6	50	−0.571 ^4^
19 August 2017	00:00	8:00	396.9	7.75	0.042	2	13	−0.138

^1^ UT; ^2^ Max value; ^3^ For all measured points; ^4^ Mean value.

**Table 3:** The summary of measuring data for PSR J1509+5531.

Date	Start Time ^1^	End Time ^1^	S/N ^2^	Relative Flux ^2^	ROT ^2^	No of close-ups	No of Points	Correlation Coefficient ^3^
10 April 2017	00:00	04:30	218.5	2.44	0,114	4	27	−0.468
8 May 2017	00:00	05:30	96.1	0.94	0,082	4	27	−0.712
13 May 2017	00:00	15:30	93.4	0.91	0,270	4	27	−0.468

^1^ UT; ^2^ Max value; ^3^ For all measured points.

## Data Availability

The data presented in this study are available on request from the corresponding author.

## Abbreviations

The following abbreviations are used in this manuscript:

GNSS	Global Navigation Satellite Systems
LOFAR	Low Frequency Array (www.astron.nl/telescopes/lofar)
POLFAR	Polish LOFAR Consortium
LOFAR4SW	LOFAR for Space Weather
EU	European Union
HBA	High Band Antennas
LBA	Low Band Antennas
ILT	International LOFAR Telescope
VLBI	Very Large Baseline Interferometry
TEC	Total Electron Content
EISCAT	European Incoherent Scatter Scientific Association
RO	Radio Occultation
TID	Travelling Ionosphere Disturbances
ISM	Interstellar Medium
RM	Rotation Measure
DM	Dispersion Measure
SNR	Supernova Remnant
S/N	Signal to Noise ratio
DSPSR	The Digital Signal Processing for Pulsars package (http://dspsr.sourceforge.net/)
AGW	Atmospheric Gravity Waves
STEC	Slant Total Electron Content
IGS	International GNSS Service
ROT	Rate of TEC
ROTI	Rate of TEC Index
IPP	Ionospheric Pierce Point
GPS	Global Positioning System
GLONASS	GLObal NAvigation Satellite System
FORMSAT	Formosa Satellite Mission
COSMIC	Constellation Observing System for Meteorology, Ionosphere, and Climate
